# Rehabilitation Decision-making for Lower Extremity Sarcoma with Undiagnosed Metastases: A Case Report

**DOI:** 10.7759/cureus.5439

**Published:** 2019-08-20

**Authors:** Mary Alice Hewelt, Christopher M Wilson, Elyse A Mazurkiewicz

**Affiliations:** 1 Physical Therapy, Beaumont Health, Grosse Pointe Hospital, St. Clair Shores, USA; 2 Physical Therapy, Oakland University, Rochester, USA; 3 Physical and Occupational Therapy, Beaumont Health, Troy, USA

**Keywords:** sarcoma, pathologic fracture, aquatic physical therapy, joint mobilization, cancer, metastases, pain

## Abstract

This case report describes the physical therapy management and clinical decision-making for a 67-year-old female patient with an initial left-hip sarcoma which subsequently metastasized. The patient had significant physical pain and emotional distress after her surgery and radiation. The patient presented to physical therapy (PT) with left hip pain and pain in the left flank and left shoulder. These issues were significantly affecting her quality of life and activities of daily living. She had undergone a previous bout of outpatient PT that did not resolve her pain. A thorough PT evaluation was completed and conservative management of the patient’s pain was initiated but she did not experience sustainable pain relief. Later it was discovered that the patient had developed spinal metastatic lesions and the pain was likely caused by a pathological fracture that was not identified upon physical examination or previous imaging. Based on this, the physical therapist chose to conduct physical therapy due to the increasing pain, and then referred her back to the physician for further evaluation of imaging results and reevaluation of the patient’s symptoms. The initial diagnosis and metastatic spread of the sarcoma had a significant negative influence on the patient’s quality of life and participation in her activities of daily living. When working with any patient with a history of cancer, metastatic disease should remain high on the differential diagnosis list and should be a focus of any new unexplained pain.

## Introduction

Soft-tissue sarcoma is a relatively rare but invasive condition occurring in both adults and children. The most common types of sarcomas in adults are undifferentiated pleomorphic sarcoma, liposarcoma, and leiomyosarcoma [1]. While smaller soft-tissue sarcomas may be treated by surgery alone, larger or more aggressive tumors often require radiation therapy and/or chemotherapy [2]. With few exceptions, metastatic disease after soft tissue sarcoma is rarely curable, although significant benefits can be found from systemic chemotherapy and palliative radiation [2].

Survivorship and quality of life after soft-tissue sarcoma are dependent on the location of the initial sarcoma and presence or absence of metastases, with increased disability noted after lower extremity sarcomas (as compared to upper-extremity sarcomas) [3]. Initial resection often involves the decision between limb sparing resection and amputation. There is no clear differentiation in the overall quality of life between these two procedures; however, increased physical disability is found following amputation. In addition to physical disability, treatment of soft-tissue sarcoma can result in detrimental effects including lymphedema, fatigue, and psychological impairments that must be addressed by rehabilitation. In addition to physical dysfunction, impaired activities of daily living and participation restrictions, rehabilitation professionals are advised to consider psychological impairment and refer patients to the appropriate interdisciplinary team member [3].

In a retrospective analysis of 233 patients with a primary soft-tissue sarcoma who did not receive chemotherapy after resection, 56% of the patients developed metastatic disease and 24% demonstrated local recurrence. Patients who did not receive chemotherapy had a higher incidence rate of metastasis [4]. This is an important consideration for physical therapists who are consulted to manage a patient’s pain and subsequent dysfunction. The purpose of this case report was to describe clinical decision-making in the multifactorial physical therapy (PT) management of medically complex patients with lower extremity soft-tissue sarcomas and newly identified metastatic disease.

## Case presentation

A 67-year-old woman was diagnosed with a high-grade undifferentiated pleomorphic sarcoma following the resection of a rapidly growing soft-tissue mass. Her symptoms began 11 months prior to diagnosis when she initially fell on her hip and felt a bump with associated pain. In the emergency room, the initial history and physical examination resulted in the diagnosis of a hematoma around her proximal femur. Her symptoms worsened over the next nine months and she experienced recurrent falls. Radiographic imaging six weeks prior to her initial cancer diagnosis did not reveal significant abnormalities. Due to the concern for a possible hip fracture, a CT scan without intravenous contrast was obtained. While the CT confirmed no displaced fracture, it indicated clinical correlation for a nonspecific left thigh tissue collection suggestive of a hematoma. An MRI was obtained after the CT and demonstrated a soft-tissue mass in the subcutaneous tissue of the left hip suspicious for sarcoma. The patient had a consultation with an orthopedic oncologic specialist one week following the MRI and underwent a radical resection of the sarcoma the next day.

The pathology report revealed a 13.5 cm superficial left hip undifferentiated high-grade pleomorphic sarcoma with 50-60% necrosis. Margins were negative, with the closest evidence of cancer being 2 mm within surgical margins. A staging chest CT one week after surgery demonstrated two tiny lung nodules with the larger nodule measuring 4 mm which indicated a recommended follow up of three to six months. Of note, there was also a small hypodense lesion in the spleen that was inadequately visualized. Tumor stage was established to be Stage III measuring 13.5 cm at its greatest dimension. The tumor, node, metastasis (TNM) staging was established to be pT2a, cN0, cM0 without evidence of lymph node involvement or distant metastases. The patient had two follow-up appointments with the surgeon who recommended a consult to medical and radiation oncology on both occassions. She had consultations at two other institutions including a sarcoma clinic, with a recommendation of adjuvant chemotherapy and adjuvant radiation. Another chest CT was performed six weeks after surgery that demonstrated the stability of the 4 mm pulmonary nodule in the right upper lobe. The tiny nodular focus along the right hemidiaphragm was also stable.

Two months after surgery, she underwent adjuvant radiation at Beaumont Hospital (Royal Oak, Michigan, United States) for six weeks receiving 60 Gray. At the onset of radiation treatments, the physician identified a palpable seroma in the posterior thigh with tenderness. A 3-6 month follow up was recommended to monitor this finding. Upon completion of an abdominal MRI, there was no suspicious adenopathy or mass lesions in the abdomen. However, there were multiple liver cysts and a stable splenic lesion suggesting hemangioma or lymphangioma. At the next post-surgical appointment four months following her initial surgery, the physician noted that the patient was verbalizing anxiety about receiving chemotherapy. She expressed the feeling that no one was listening to her and that she did not want to proceed with chemotherapy. The physician encouraged the patient to follow this recommended course of action and proceed with the provided referrals. Upon conclusion of the appointment, she agreed to proceed but subsequently did not receive this adjuvant chemotherapy.

**Figure 1:**
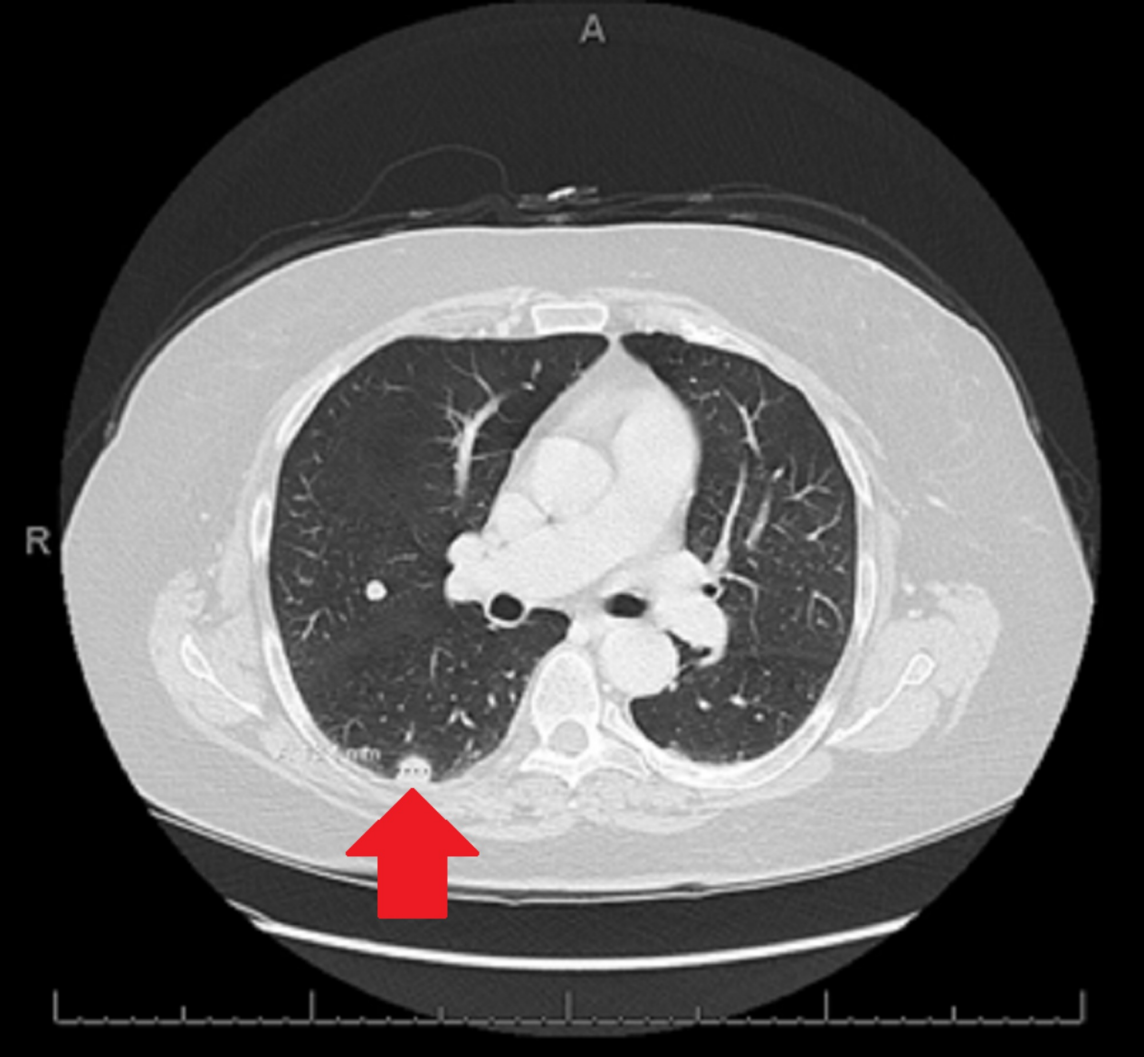
Pulmonary nodule via CT scan Interval enlargement of pulmonary nodules; the example here is the superior segment of right lower lobe (previously 0.6 cm, now 1.3 cm).

Another chest/abdomen/pelvis CT was performed four months following surgery which showed the development of four new pulmonary nodules which were concerning for metastasis (Figure 1). In addition, a large (approximately 13.1 x 9.9 x 10.3 cm) fluid collection within subcutaneous tissue was observed in the left lateral aspect of the left hip. The differential diagnosis list for this finding included seroma, chronic hematoma or abscess. Two stable hypodense lesions were noted, one each in the liver and spleen. The patient followed up with medical oncology from this institution after this finding.

Clinical findings

**Figure 2:**
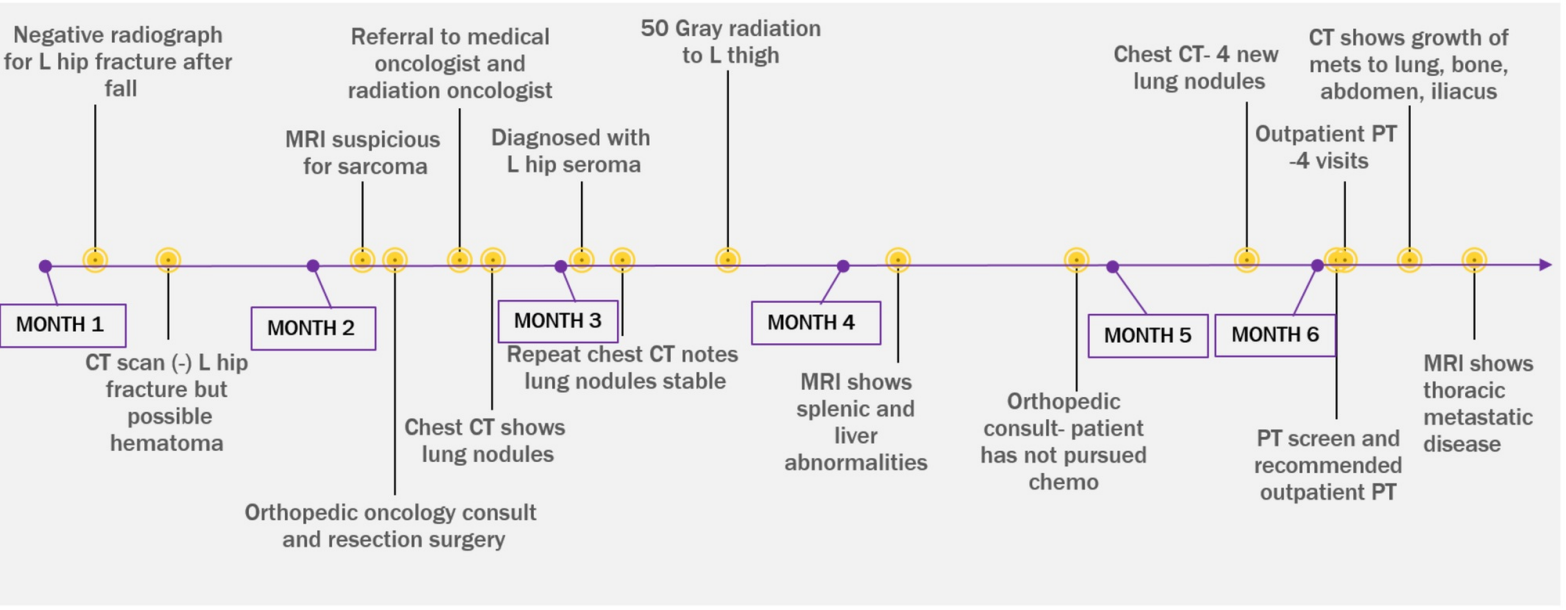
Cancer journey timeline L = left; CT = computerized tomography, MRI = magnetic resonance imaging; PT = physical therapy

This patient presented to physical therapy six months after her initial sarcoma diagnosis and surgery (Figure 2). She presented with left shoulder, left flank and left thigh pain. She reported that her past medical history included the left hip sarcoma resection with adjuvant radiation therapy (RTx). She previously attended physical therapy at another facility for treatment of the shoulder pain which began after the course of RTx due to worsening pain. She was seeking care from Beaumont St. Clair Shores Physical Therapy due to a fresh onset of left flank pain and increasing left hip pain. Her physician recommended the aquatic therapy available at our facility. She did not have a prescription with her at the time of screening but stated that her previous prescription was for the shoulder only. Based upon the initial screening for rehabilitation needs, a prescription for lymphedema of the left lower extremity, abdomen, hip, flank, and shoulder pain was sent to her referring physical medicine as well as a rehabilitation (PMR) physician. The initial referral provided by the referring physician did not specify any precautions, contraindications, or other restrictions.

Subjective History

The patient stated she lived in a home with her husband and developmentally disabled adult son. Prior to the onset of her symptoms and deficits eight months ago, she exercised regularly using a treadmill and exercise bike in her home. She was very distressed about the recent weight gain of more 40 pounds since surgery and RTx. She stated that the treatment at the other PT clinic included exercises for the shoulder, 5-10 minutes of “massage” on the shoulder area and 10 minutes of heat at the end. It was during the course of this previous episode of care that the left flank pain began. The left hip pain had been ongoing since surgery and RTx, and had worsened recently. She expressed significant anxiety about the course of her cancer survivorship with regards to determining who to use for medical oncology and what course of treatment to take. She had received consultations from multiple institutions but was unable to decide on a course of action. She also stated that she felt as though no one was listening to her concerns. She denied alcohol or illicit drug use but was a former smoker (25 years) having quit 16 years ago. Her current pain management regimen was 1 tablet of oxycodone (Percocet 10/325 mg) two times per day (despite being prescribed every six hours as needed for pain) and an acetaminophen (Tylenol 500 mg) tablet as needed. She avoided the use of her prescribed cyclobenzaprine (Flexeril) because it made her feel like a “zombie” and resulted in an increase in muscular spasms.

The patient had a past medical history significant for resection of a benign tumor in the pituitary gland causing acromegaly. She also had recurrent episodes of vertigo that had been successfully treated with medication for nearly ten years. She had hypertension that was also managed with medication. She had a right knee meniscus injury approximately three years ago that was resolved with physical therapy intervention. Finally, she had a history of cholecystectomy.

Tests and Measures

The patient’s subjective functional deficits of greatest concern were pain (7-10/10), difficulty with sleep, standing to wash dishes, dressing, grooming and sitting tolerance so she could watch television with her son. Evidence-based subjective assessment tools of Patient-Specific Functional Scale (PSFS), Upper Extremity Functional Index (UEFI) and Modified Oswestry Disability Index (ODI) were used. Although the patient’s series of events did not allow for reassessment of these measures, it is worthwhile to note that the minimally clinically important difference (MCID) or minimum important difference (MID) for each of these measures have been established.

Stratford developed the PSFS in 1995 for use in patients presenting with various musculoskeletal disorders, having varying levels of independence and established the minimum detectable change (90% confidence interval) for the average of 3-5 activities to be two points; and for a single activity, three points [5]. In 2014 in a cohort study of 1,708 consecutive subjects, Abbott presented the MID for the PSFS average score (1.3-2.7), UEFI (6-11), Lower Extremity Functional Scale (LEFS) (9-16), Neck Disability Index (NDI) (-14) and ODI (-12) when compared to the 15-point Global Rating of Change (GROC), the recommended reference standard for studies of MID [6-7]. Horn established the PSFS to be reliable, valid and responsive to change in knee, lower back, and neck dysfunction [8]. Hefford further established the reliability, validity, and responsiveness of the PSFS with upper extremity dysfunction [9]. Chesworth found that the UEFI was a reliable measure (Intraclass correlation coefficient (ICC)=0.94) [10]. The MCID for the UEFI was found to be between 6-7 depending on which version of the UEFI was used. Chesworth also identified that the MCID was smaller for those with a problem in their dominant arm as compared to a problematic non-dominant arm. Schwind et al. noted that there is not one definitive MCID for the Modified ODI, and that a 30% change or a 5, 6, 10 and even a 17-point change may be the MCID [11]. Although this test does not have a clear MCID, it is very commonly used in patients with back pain and is frequently referenced and understood by insurance companies in helping to determine reimbursement.

Functional and objective deficits were identified during the examination and resulted in patient-centered prognosis, goals, and interventions (Table 1). Goals were also established for the self-reported UEFI and Modified ODI that are noted above. In addition, the therapist and patient developed and mutually agreed upon PSFS functional goals including the following: (1) improved quality and quantity of sleep, (2) increased sitting tolerance to improve quality of life with family activities and (3) decreased difficulty with dressing, grooming, and household chores.

**Table 1:** Summary of impairments, goals, and prognosis Key: ROM = range of motion; flex = flexion; abd = abduction; t/o = throughout; w/ = with; L = left; R = right; UE = upper extremity; MMT = manual muscle test; LTG = long term goal; SLR = straight leg raise; w/o = without; STG = short term goal: PSFS = Patient Specific Functional Scale

Impairments	Initial Status	Prognosis / Goals	Intervention
ROM	Shoulder flexion: L 70° with pain, R 161°. Shoulder abduction: L 58° with pain, R 175°. Thoracic spine:25% decreased throughout. pain with L rotation and R sidebending	LTG to increase shoulder flex and abd 150°, hip flex and SLR w/o pain, thoracic mobility 50% w/o pain	Joint mobilization, passive/active assistive/active ROM, flexibility exercises, and stretching
Strength	MMT: L UE unable to tolerate MMT; R UE 4 to 4+/5 except shoulder extension 5/5	STG to tolerate MMT of L UE; LTG to increase B UE 4+/5	Aquatic and land-based exercise program providing active, assistive, and resistive exercises for strength, power, and endurance training
Edema	Thigh circumference : L thigh = 4 cm; R thigh = 4.8 cm	LTG: Reduce L thigh circumference by 0.8 cm	Manual lymph drainage, ROM exercises and (if required) compression bandaging
Upper Extremity Functional Index	Initial Score: 26	Goal Score: 42	*See interventions above
Modified Oswestry Disability Index	Initial Score: 78%	Goal Score: 50%	*
Sleep	Revised PSFS Score: 9	Goal Score: 4 w/ ↓ pain medication	*
Doing Dishes	PSFS Score: 0	Goal Score: 5	*
Dressing	PSFS Score: 2	Goal Score: 6	*
Grooming hair	PSFS Score: 1	Goal Score: 6	*
Sitting Tolerance	PSFS Score: 1 to be able to watch television with son	Goal Score: 6	*

Evaluation and Clinical Decision-making

Based on her complaints of positioning difficulty during RTx and clinical findings, evidence demonstrated that the onset of shoulder pain was a result of mechanical dysfunction. She discontinued PT at the previous facility due to the onset and continuation of flank pain and the increasing left hip/thigh pain. It is also reasonable to consider that exercises or activities during the previous PT could have contributed to the new onset of pain. Her PMR physician advised aquatic therapy for an alternative pain management option.

At that time, the major concern was continued surveillance of lung nodules with a “concern for metastasis” noted in the imaging report approximately two months prior to PT examination. Despite lack of evidence of osseous metastasis, there was awareness created by the therapist that it was a small but possible outcome. Therefore, treatment options needed to focus on optimal mobility and pain control utilizing minimal and controlled force as needed, which would decrease the risk of fracture/dislocation if osseous metastasis occurred.

Of additional concern was the patient’s indecision regarding selection of a medical treatment plan and provider. This caused a significant delay in receiving any medical intervention that may have had a significant effect on her disease process. Based on the information obtained from numerous sources, the patient was quite frustrated and anxious, feeling as though no one was listening. Yet the medical documentation indicated that she was being provided a clear and consistent recommendation to have adjuvant chemotherapy. A significant barrier to her accepting this treatment plan seemed to be her concern for her son’s well-being in the event she was unable to help him.

The patient’s emotional and psycho-social concerns warranted referral to social work, psychology/psychiatry, and case management. Upon inquiry whether she had access to any of these services, she stated that she received counseling during home visits but she had been unable to sit long enough to tolerate a session. She had tried to utilize the social work services at an outside institution, but was extremely frustrated that she was advised over the phone to seek anti-anxiety medication. The therapist and patient also discussed community and social work resources that may be available for her son.

Physical Therapy Prognosis

The patient’s deficits included pain, decreased ROM and strength, and lymphedema of the abdomen and left thigh. These resulted in limitations with functional activities affecting her ability to care for herself and her family. It was anticipated that deficits could be resolved with a treatment plan that included: (1) education for positioning, body mechanics, activity modification, joint protection and postural training (2) aquatic therapy to provide an exercise medium that would reduce the effects of the recent weight gain that was making it more difficult for her to move (3) neuromuscular re-education to improve movement patterns to reduce pain (4) manual interventions to also reduce pain, increase blood flow, improve joint mobility and flexibility and (5) manual lymph drainage to facilitate circumferential reduction in the abdomen and left upper leg.

Therapeutic interventions

After the initial evaluation, the patient received three visits of physical therapy to attempt to reduce the patient's pain and improve her impairments and functional limitations. When these did not demonstrate improvements, the patient was referred back for further diagnostic and medical workup. 

Visit 1: Interventions and Outcomes

Visit 1 of treatment included aquatic exercises for ROM, endurance, stretching, and pain control for approximately 30 minutes. The manual treatment that was performed on-land included techniques focusing on pain control and mobility such as upper quadrant soft tissue mobilization (STM) to her periscapular muscles, functional massage to the upper trapezius, supraspinatus, subscapularis and latissimus, and lower quadrant STM to the left hip scar tissue, iliotibial band, hamstrings, quadriceps, gluteals, quadratus lumborum, and thoracic and lumbar paraspinals. Education provided included energy conservation, activity modification and positioning for pain control.

The patient verbally reported that her pain was reduced from 4/10 to 2/10 after her aquatic exercises, but increased after getting dressed. After her on-land treatment, her pain was reported at 3/10. She had visible improvements with upper extremity range of motion (ROM) and decreased tissue tension in the upper and lower quadrants. She demonstrated a good understanding of the educational interventions provided to her.

Visit 2: Interventions and Outcomes

Visit 2 of treatment included aquatic exercises as established on Visit 1. She also participated in land-based treatment which focused on her thoracic symptoms. She stated she had less pain today (3/10) but felt like a spasm could happen at any moment. She tried a whole Flexeril tablet the night before treatment Visit 2, but felt no relief of symptoms. Her pain was described as cramping inferior to the scapula. Interventions included assessment of thoracic and rib alignment, that identified elevated left ribs 6-10. Also, the T6-T10 segments were found to be positioned in right rotation and right side bending.

The mobilization techniques chosen were based on the Nordic System of Orthopedic Manual Therapy (OMT) and Mulligan’s SNAGs, NAGs, and Mobilizations with Movement techniques [12-13]. Nordic OMT includes identifying a need for mobilization after the loss of joint movement, pain with movement, and pain with specific functional activities. It also requires identifying the pain-free articular translatoric movement for the glide to be performed that improves pain and movement. In addition, active patient movement through a specified range of motion and pain-free over-pressure are utilized. Reassessment of the movement pattern identifies the outcomes of the treatment.

Mobilizations included left ribs 6-10 utilizing Mobilization with Movement (MWM) techniques as described by Mulligan [13]. These MWM techniques generally include Natural Apophyseal Glides (NAGs) and Sustained Natural Apophyseal Glides (SNAGs) which is the “concurrent application of sustained accessory mobilization applied by a therapist and an active physiological movement to end range applied by the patient” [12]. These techniques included depression with expiration, T6-10 anterior-superior NAGs and T6-10 anterior-superior with side bending SNAGs. Soft tissue mobilizations for bilateral thoracic paraspinal muscles were additionally performed. Education included diaphragmatic breathing with counting out loud during exhalation to facilitate the glottis, diaphragm and pelvic floor; proper sitting posture with emphasis on symmetry, avoiding positions of coupled rotation and side bending into the position of dysfunction, and performing straight plane active range of motion thoracic flexion/extension, bilateral rotation and bilateral side bending in pain free range.

The patient demonstrated better motor control during aquatic exercises as measured by therapist clinical observation. She advanced to bilateral lower extremity standing straight leg raise exercises and increased reps for all upper extremity exercises. She reported that her pain went down to a 2/10, while in the pool but that it came back up to a 4/10 after dressing herself (specifically with putting on her bra).

The T9 motion segment remained slightly rotated to the right while all other segments and ribs demonstrated improved alignment as measured by therapist palpatory assessment. The patient stated she was slightly achy after mobilizations but her pain was improved (2/10) and her paraspinals muscles no longer felt like they were going to spasm. She demonstrated understanding and competence with the extensive education in diaphragmatic breathing, posture, positioning and mobility exercises with the focus of pain control and facilitating improved movement patterns.

*Visit 3*:* Interventions and Outcomes*

The patient subjectively reported that she had to take a pain pill that morning due to “a lot” of pain overnight because she was trying to be more active over the weekend with walking more. Her mid-back pain was 5/10 and left shoulder pain was 3/10. The patient’s aquatic exercises were continued as previously established.

The patient was able to tolerate increased repetitions for all shallow water exercises. Handheld paddles designed to provide minimal water resistance during aquatic movement were utilized during upper extremity exercises. She ended the aquatic component of her treatment session with decreased pain (3/10) in both locations.

Upon initiation of on-land treatment, the patient stated that she did not want to proceed with additional treatment that day. She stated she had received the results of her most recent CT scan and that they were “not good.” The patient made no mention of test results prior to or during the aquatic treatments on this date. She expressed significant frustration stating that she was not receiving definitive guidance from her physicians. She reported that she was attempting to pursue ongoing cancer care with an outside institution which provided one of her consultations, but was having difficulty scheduling appointments.

**Figure 3:**
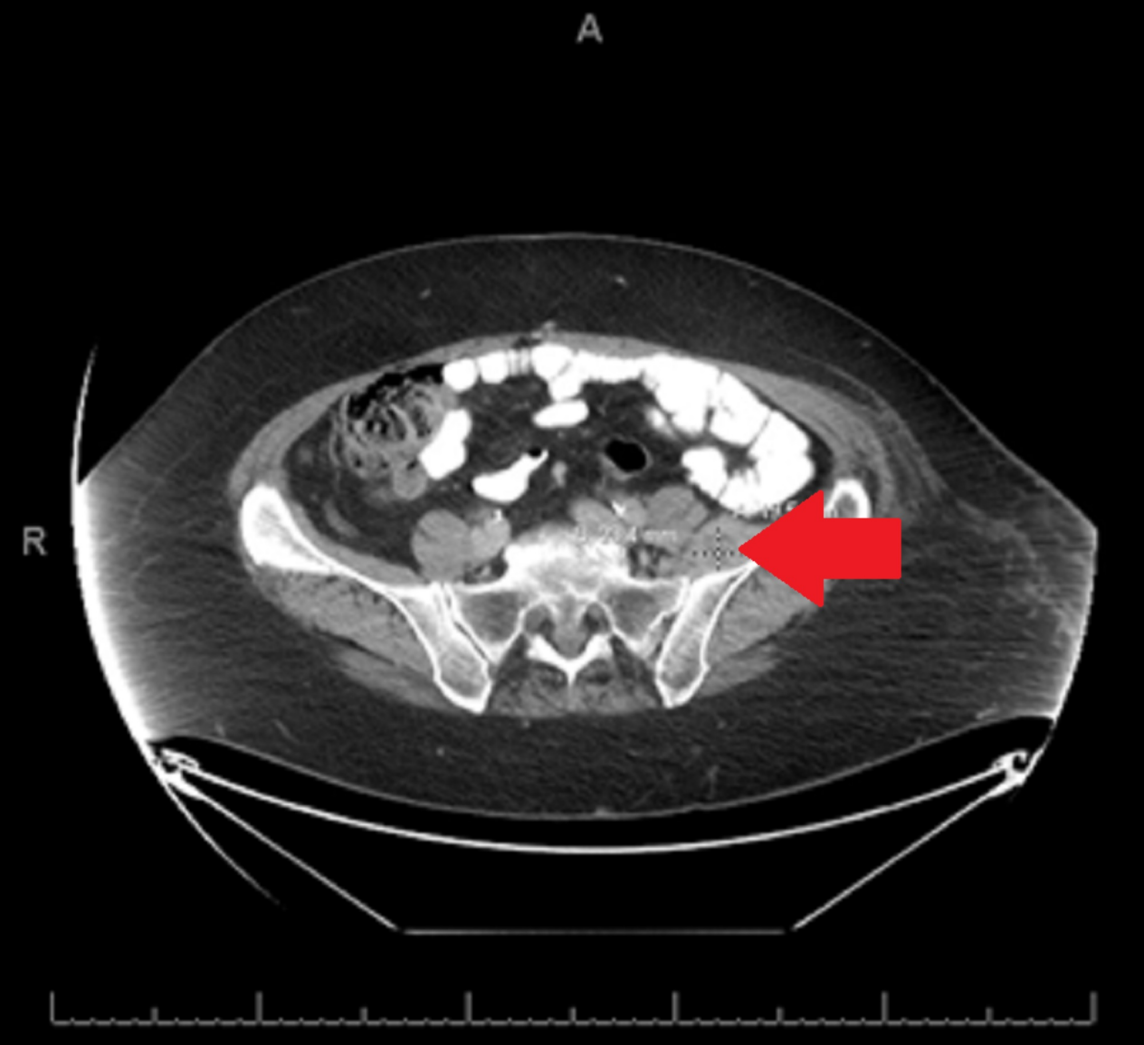
Mass near the left iliacus muscle via computerized tomography Soft tissue mass in the left iliacus muscle measuring 1.9 cm x 2.3 cm

**Figure 4:**
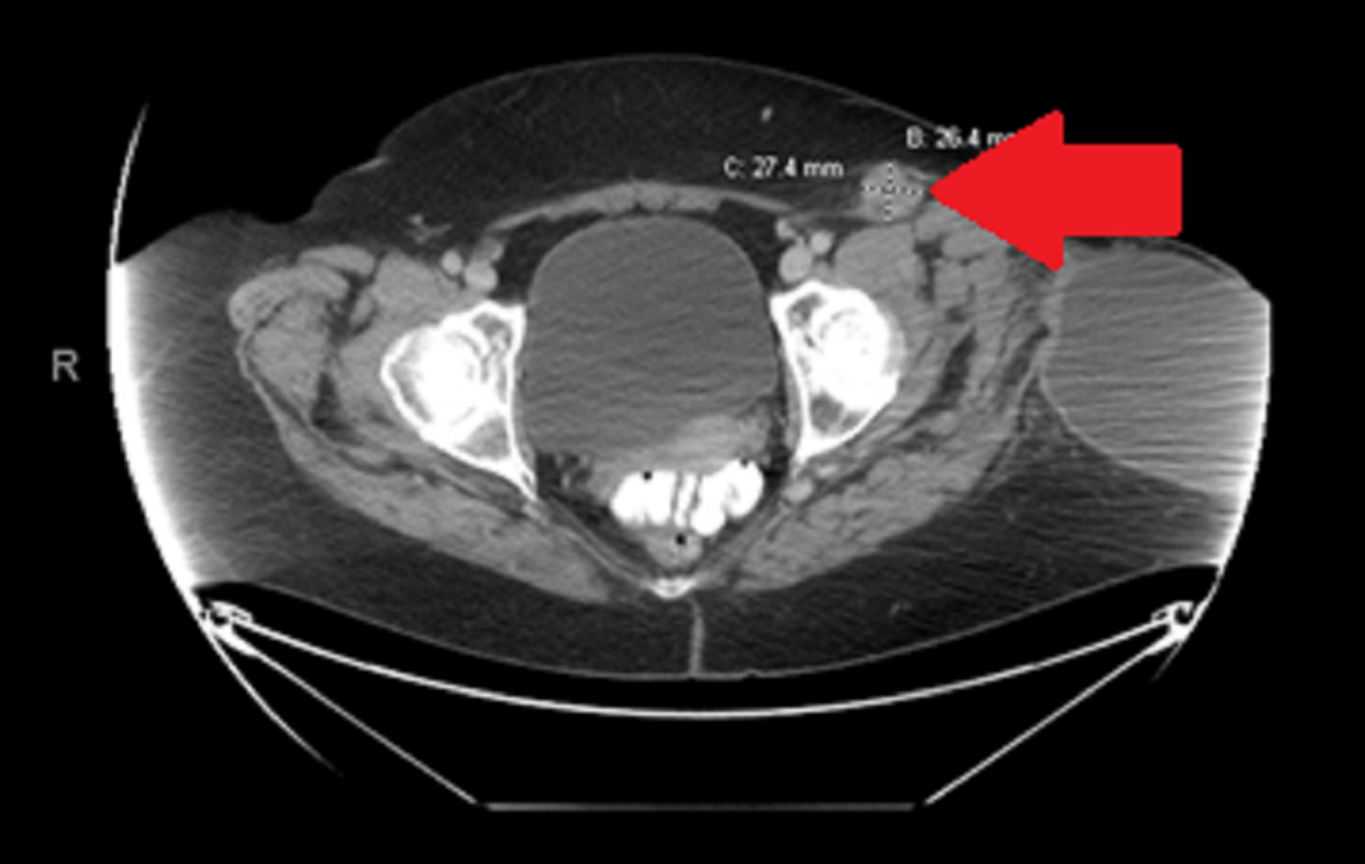
Enlarged left inguinal lymph node via computerized tomography Left inguinal region pathologic lymph node measuring 2.7 cm x 2.6 cm

**Figure 5:**
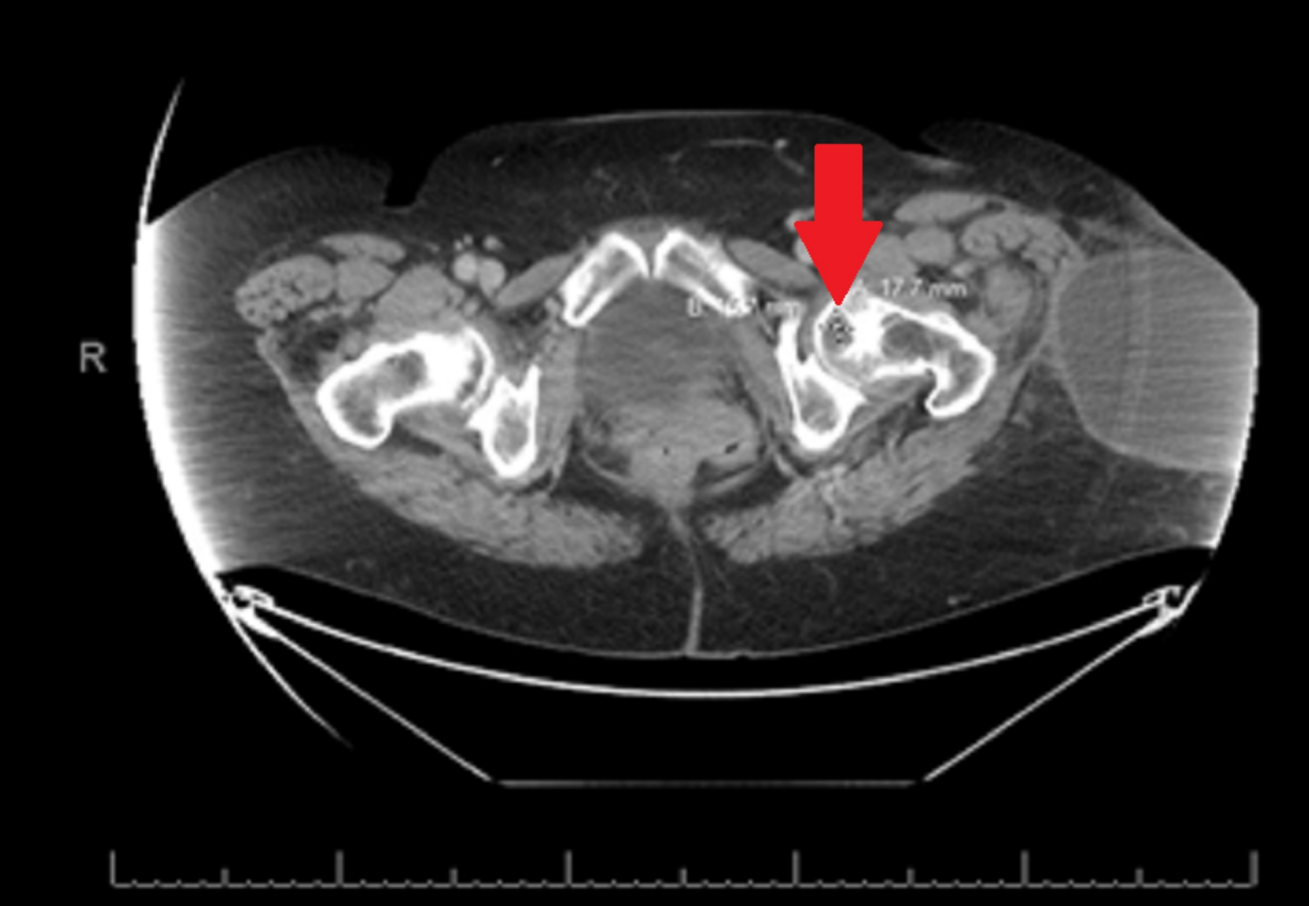
Lytic lesion of the left femoral head via computerized tomography A lytic lesion in the left femoral head measuring 1.7 cm x 1.5 cm

**Figure 6:**
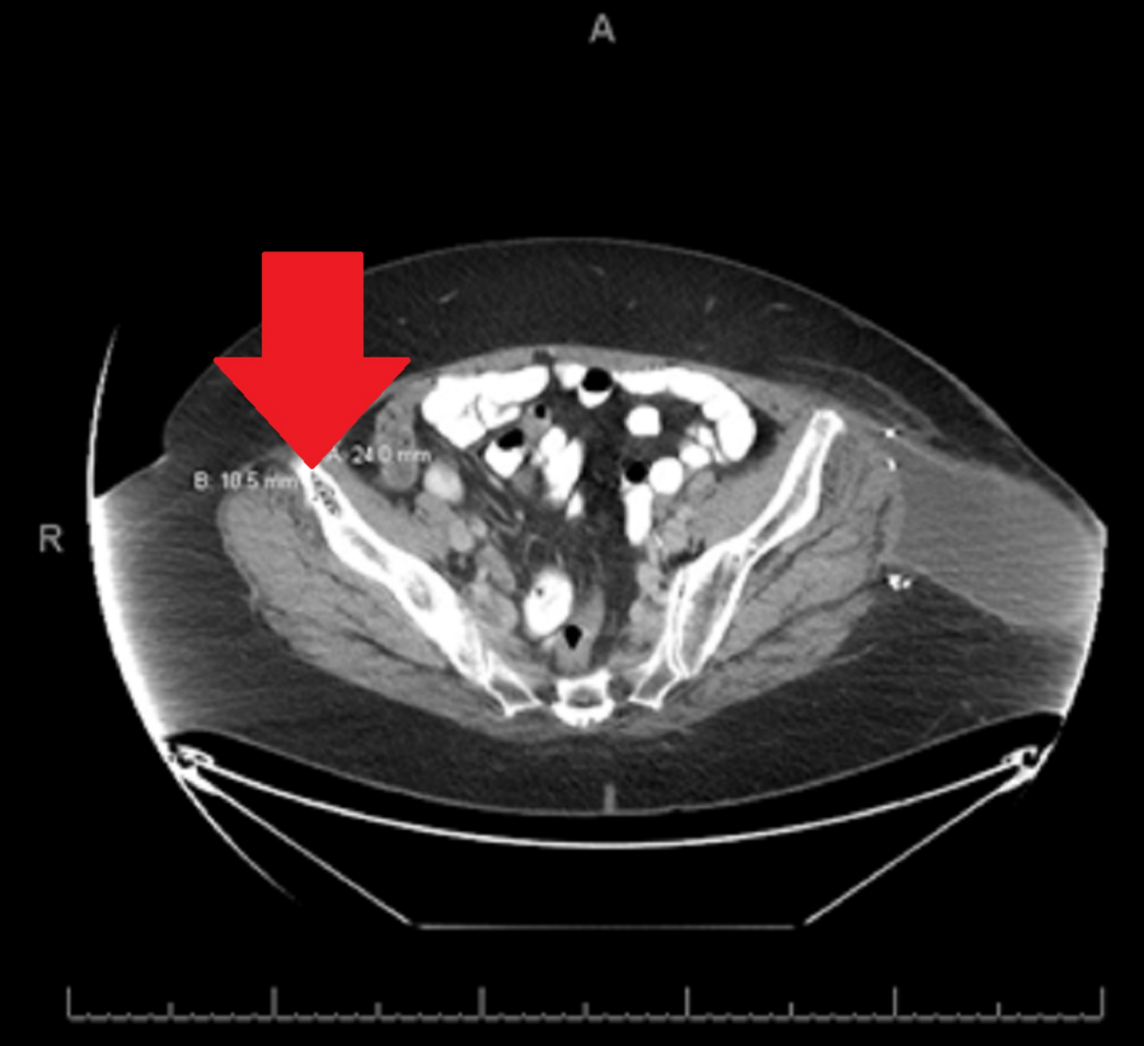
Right iliac crest lesion via computerized tomography Right iliac bone lytic lesion measuring 2.4 cm x 1.0 cm

The evaluating therapist reviewed the recent results of the CT of the chest/abdomen/pelvis. Results included multiple new masses in the abdomen/pelvis including the left iliacus muscle and an enlarged left inguinal lymph node (Figures 3-4). In addition, bony metastases were noted to the left femoral head and right iliac crest (Figure 5-6). Finally, a T7 vertebral lesion was found measuring 2.7 cm x 2.2 cm with extension into the spinal canal abutting the spinal cord (not pictured).

The evaluating therapist had an extensive conversation with the patient by utilizing active listening to her extraordinary distress. This included notation of severe pain that was not being managed effectively. While her subjective assessment of her pain was 2-5/10, she was concerned about the amount of pain medication required to decrease her pain. This was leading to increased anxiousness since she could not tolerate performing her daily tasks as well as her family’s personal finances and medical/social work management for her son with developmental disabilities. She was acutely aware of her potential mortality and was trying to get her affairs in order for her son, but she was so stressed from her pain she could not function. She stated her husband was not a source of support or assistance. She stated repeatedly that she "is at the end of my rope; it would be better if I just shoot myself. I wouldn't do it; but that is how bad I feel." Of note, numerous risk assessments by this institution’s physicians and this writer were previously negative for suicidal ideations. Upon inquiry, the patient stated that when she spoke with the social worker from the sarcoma clinic three days prior, she was recommended anti-anxiety medications but had not acted upon this advice. The therapist encouraged the patient to seek resources through these disciplines for securing her son's future and addressing her own mental health.

The patient was also advised on the importance of following up with medical oncology and her referring physician to determine if it was in her best interest to continue with PT at this time based on these new findings. The patient was encouraged to go to the emergency room (ER) to seek medical attention to control her pain and to obtain further testing if appropriate. She was very discouraged by the thought of not being able to continue with her pool exercises since it was the only thing she looked forward to, but verbalized understanding of the importance of receiving medical clearance with regards to the most recent test results. The therapist was provided with options and advised that the patient should go to the emergency room for care based on her emotional and medical issues. She left the facility with a plan to seek further medical attention after she got affairs in order for her son.

The treating therapist also provided advocacy by contacting her institution’s palliative care team for information regarding palliative care options for this patient. The patient was subsequently called to reinforce this recommendation. Later that evening, the patient presented to the emergency room and was admitted with consults to medical oncology and orthopedics.

Outcomes

**Figure 7:**
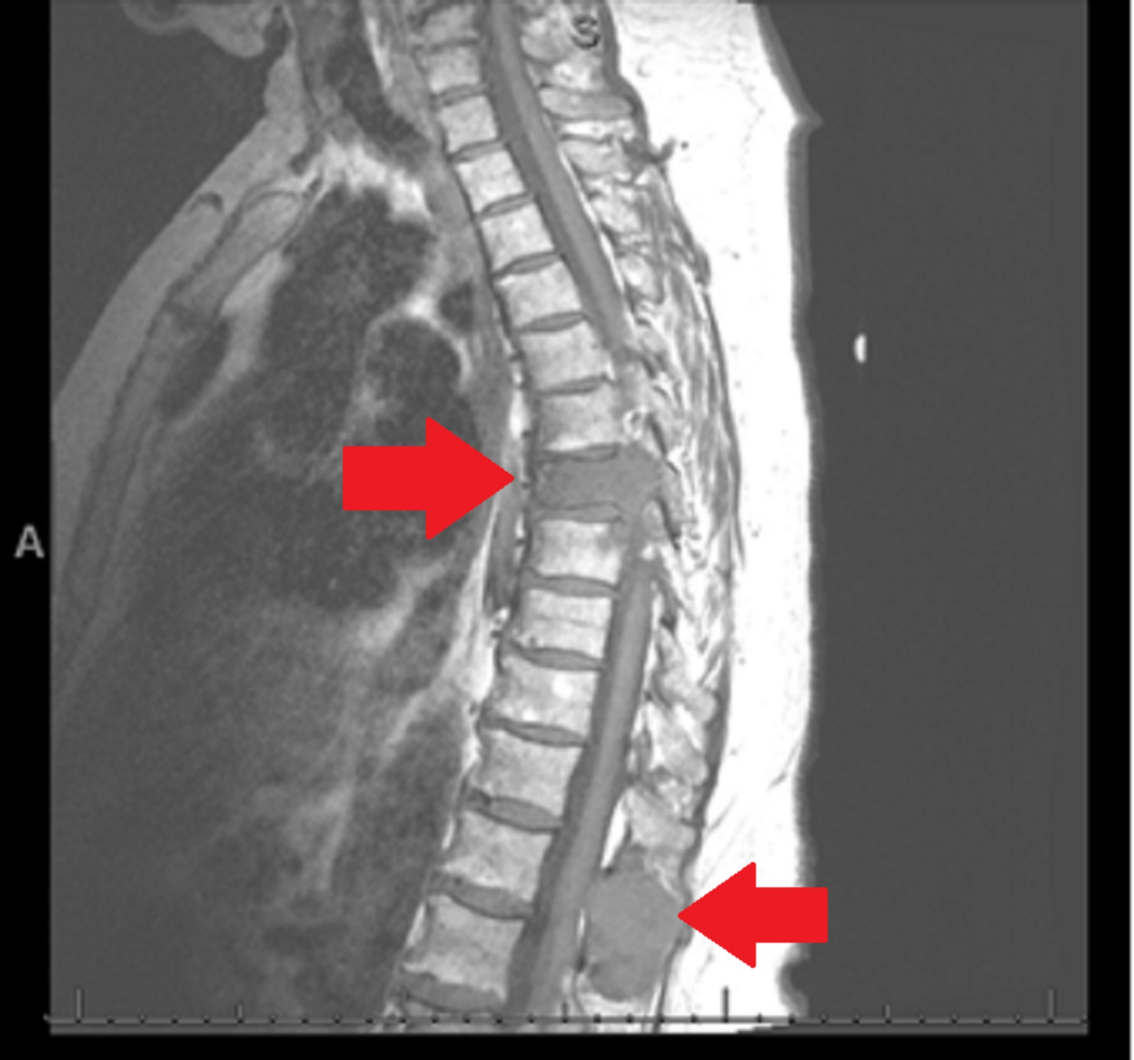
Lesions of T7 and T12 via magnetic resonance imaging T1 weighted image of thoracic spine demonstrating pathologic lesions of T7 and T12

**Figure 8:**
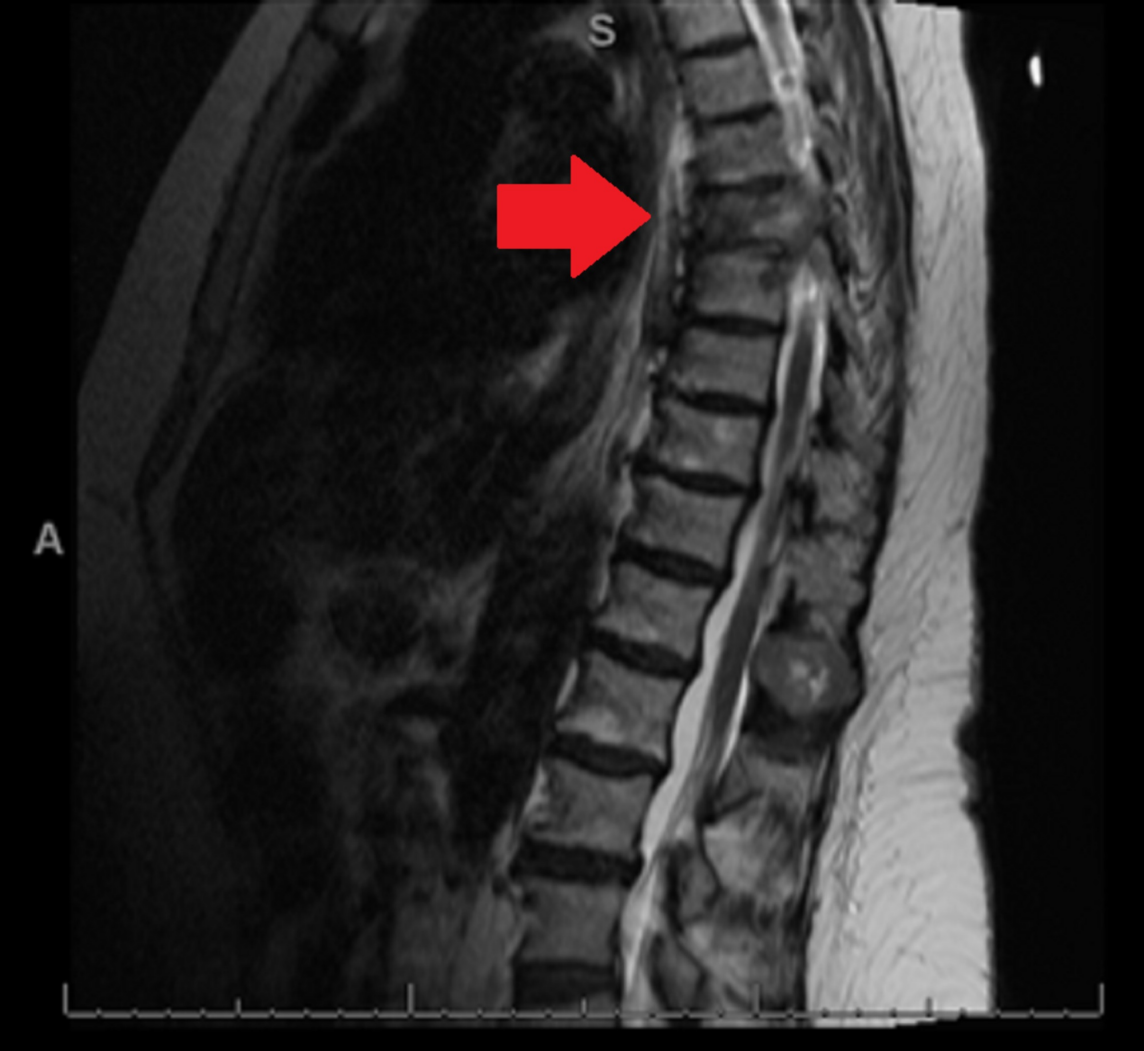
Thoracic spine lesions via magnetic resonance imaging T2 weighted magnetic resonance image demonstrating thoracic spine lesions. Red arrow demonstrating T7 vertebral body pathologic fracture and collapse.

Her hospital course of care included an MRI that indicated extensive metastatic disease. The dominant T7 vertebral lesion demonstrated possible pathologic vertebral fracture with extension into the posterior elements and apparent near-complete effacement of the left neural foramen with a likely compromise of the exiting nerve root. An abutment of the ventral spinal cord without intrinsic spinal cord signal abnormalities was identified. Some degree of posterior cerebrospinal fluid space remained. Evidence of anterior and left lateral epidural invasion at this level was noted, as well as the T12 lesion exerting a mass effect on the dura, which may have been invading it (Figures 7-8).

The patient was advised by her attending physician that her best course of action was to transfer to an outside institution for surgical intervention to stabilize the spine, and subsequent medical management through the sarcoma clinic. She was transferred to that institution three days after admission.

Upon follow-up, the patient reported that she received surgical intervention on her thoracic spine. However, she was unclear of the details. She was transferred to a skilled nursing facility near her home to be more conveniently located to her family. Additional follow-up has been unsuccessful as at the time of writing this case report.

## Discussion

This case report described the initial clinical presentation and clinical decision making with a patient with metastatic sarcoma. The patient’s primary complaint was related to multiple areas of pain and dysfunction, including flank pain. In this case, the therapist provided an initial treatment plan with information that included no bone metastases. However, upon repeat scans after the third visit for PT treatment, additional information was provided that indicated that the patient’s pain may have been related to a previously undiagnosed metastatic lesion as opposed to joint or muscular dysfunction. This patient presented with a loss of uniaxial and multiaxial movement in numerous thoracic spinal segments and ribs, in addition to shoulder and hip deficits. Based on the patient’s history and experiences provided during the interview and the examination results, positional faults and hypomobility were realistic conclusions for the source of the thoracic symptoms. Metastatic disease with an osseous component in the spine was also on the differential diagnosis list. However, repeated radiological studies had demonstrated no spinal involvement, but only lung nodules with a “concern for metastasis” prior to the PT evaluation. In light of the relative risk of skeletal metastases, mobilizations were chosen and administered with great care and of a low grade of movement with emphasis on patient participation in a similar fashion that she would do on daily basis (e.g. breathing, pain-free movement patterns). The purpose was to provide pain relief at the time of treatment and to facilitate improved movement patterns to reduce intensity and duration of future episodes of pain, which was temporarily achieved. This is not only consistent with rehabilitative goals, but also palliative care goals.

There is little to no specific guidance as it relates to determining the risks vs. benefits of spinal mobilization in patients with a history of cancer with no clear diagnosis of spinal metastases. In this case, the patient demonstrated some initial pain reduction and subjective improvement; however, this was transient. For this patient, the metastatic lesions were identified early in this episode of care and the therapist was able to make informed decisions to pursue emergent referral for further diagnostic workup and medical management. Often physical therapists are at a disadvantage when conclusively ruling out skeletal metastatic lesions as it is not historically within their scope of practice to order imaging tests. In a large systematic review of the evidence on malignant spinal metastases, Sutcliffe et al. found that early diagnosis of spinal metastasis and vertebral collapse still rely heavily on CT or MRI [14]. Other prognostic factors include tumor burden, presence of metastases at initial diagnosis, pain, and weakness.

Rose and Buchowski noted that pain is the most common symptom found with vertebral metastases [15]. This presents a dilemma for physical therapists, especially those in outpatient orthopedic settings since a high number of patients with varying cancer histories are encountered, and nearly all patients will present with some pain. Oftentimes, this pain is reproducible with movement, compression, and could be alleviated by distraction, which are classic clinical presentations of a treatable physical therapy diagnosis. Based on the absence of a clear diagnosis of spinal metastatic lesions, two key areas may be beneficial for therapists to consider in the patient with a history of cancer. This includes the presence or absence of a defined, specific precipitating cause of the pain (e.g. trauma, exertional activity) as well as the patient’s sustained response to physical therapy treatment. In this case, the patient did not have a clear onset or precipitating cause and did not demonstrate expected improvement in pain with the physical therapy treatment plan. In the absence of a clear diagnosis, these two clinical findings may lead the therapist to further discuss imaging with the physician.

Use of joint mobilizations in the presence of diagnosed metastatic bone disease is generally contraindicated, but there is little guidance for therapists in advanced cancer without a clear diagnosis of the cause of pain. In a number of manual therapy schools of thought, the analysis of spinal mobility dysfunction and provocation/alleviation testing is a component of the diagnostic process. As defined by the American Physical Therapy Association, these interventions require constant evaluative skills such as that which can be provided only by a licensed physical therapist [16]. In addition, there is further diagnostic value in a condition that may not be improving (or worsening) when improvement is expected, thereby ruling out conditions that would have been corrected by the movement dysfunction. It is clear that continued research is required in the area of manual therapy in patients with advanced cancer without a diagnosis of bone metastases, especially when the symptoms do not respond as anticipated or when a planned cancer treatment regimen was not occurring as expected (i.e. her physician recommended chemotherapy but the patient did not receive it).

As this is a case report, no generalities may be gleaned from the findings; however, this article brings to light a dilemma in physical therapy practice. The therapist’s limited ability to utilize and order diagnostic imaging for the examination of orthopedic stability may increase the risk of conventional treatments causing harm. Several states in the United States are initiating legislation to include ordering and interpreting of diagnostic imaging into the physical therapist’s scope of practice. This area of practice would benefit from a long-term plan of developing diagnostic algorithms for the clinician without access to imaging to be able to best identify the risk of those at high risk of metastatic disease.

## Conclusions

This patient initially presented with common musculoskeletal aches and pains while being concurrently treated for sarcoma. Although there was no evidence of clear metastatic disease upon imaging or physical examination, the patient did not initially demonstrate substantial carryover of pain relief after physical therapy interventions, including gentle manual therapy/mobilizations. This finding, in addition to subsequent imaging results, required the physical therapist to discontinue treatment and advocate for the patient obtaining emergent care for psycho-social distress and spinal metastases.
